# Integrative analysis reveals clinical phenotypes and oncogenic potentials of long non-coding RNAs across 15 cancer types

**DOI:** 10.18632/oncotarget.9037

**Published:** 2016-04-27

**Authors:** Ze-Lin Wang, Bin Li, Stephen R. Piccolo, Xiao-Qin Zhang, Jun-Hao Li, Hui Zhou, Jian-Hua Yang, Liang-Hu Qu

**Affiliations:** ^1^ Key Laboratory of Gene Engineering of the Ministry of Education, Sun Yat-sen University, Guangzhou, China; ^2^ State Key Laboratory for Biocontrol, Sun Yat-sen University, Guangzhou, China; ^3^ Department of Biology, Brigham Young University, Provo, Utah, USA; ^4^ Department of Biomedical Informatics, University of Utah, Salt Lake City, Utah, USA; ^5^ Department of Surgery, Li Ka Shing Faculty of Medicine, University of Hong Kong, Hong Kong

**Keywords:** lncRNA, expression profile, prognostification, somatic copy number alteration

## Abstract

Long non-coding RNAs (lncRNAs) have been shown to contribute to tumorigenesis. However, surprisingly little is known about the comprehensive clinical and genomic characterization of lncRNAs across human cancer. In this study, we conducted comprehensive analyses for the expression profile, clinical outcomes, somatic copy number alterations (SCNAs) profile of lncRNAs in ~7000 clinical samples from 15 different cancer types. We identified significantly differentially expressed lncRNAs between tumor and normal tissues from each cancer. Notably, we characterized 47 lncRNAs which were extensively dysregulated in at least 10 cancer types, suggesting a conserved function in cancer development. We also analyzed the associations between lncRNA expressions and patient survival, and identified sets of lncRNAs that possessed significant prognostic values in specific cancer types. Our combined analysis of SCNA data and expression data uncovered 116 dysregulated lncRNAs are strikingly genomic altered across 15 cancer types, indicating their oncogenic potentials. Our study may lay the groundwork for future functional studies of lncRNAs and help facilitate the discovery of novel clinical biomarkers.

## INTRODUCTION

More than 80% of mammalian genome sequences are transcribed, but a major proportion of the transcripts do not code for functional proteins [1–3]; these transcripts are termed non-coding RNAs (ncRNAs). These ncRNAs can generally be divided into two classes based on their length: short ncRNAs (<200 nt) and long ncRNAs (lncRNAs, >200 nt). Short ncRNAs include miRNAs, which have been extensively studied in the context of cancer during the past decade [4–7]. By contrast, the functional role of lncRNAs in cancers remains largely unknown, although their number far exceeds that of miRNAs. An increasing number of studies have shown that lncRNAs may play important regulatory roles in gene expression at various levels, including at the epigenetic, transcriptional and post-transcriptional levels, and therefore they may affect the development and progression of cancer [8–11].

Recent advances in high-throughput technologies (e.g., RNA-Seq and microarray) have provided comprehensive ways to characterize the genomic profiles of lncRNAs. Indeed, the application of these approaches has revealed that the aberrant expression of specific lncRNAs may also act as biomarkers for cancer diagnosis and prognosis in glioma [12, 13], prostate cancer [14] and liver cancer [15]. In addition, through the integration of bioinformatics analyses of lncRNA SCNAs and expression profiles, a few lncRNAs with oncogenic activity have also been reported in human cancers. These include the PVT1 amplification in combination with MYC [16], FAL1 amplification and its oncogenic role in ovarian cancer [17], and PCAN-R1/2 amplification in the proliferation of prostate cancer cells [18]. However, interpreting the potential roles of thousands of lncRNAs in tumorigenesis across diverse cancer types remains a daunting challenge.

It has been widely accepted that cancer is fundamentally a genetic disease. Cancers of disparate organs may share common molecular features, whereas, conversely, cancers from the same organ can also be markedly distinct. For example, p53 mutations are common in serous ovarian, serous endometrial and basal-like breast cancer, all of which share a global transcriptional signature that involves the activation of similar oncogenic pathways [19, 20]. Such examples illustrate the importance of performing comprehensive molecular profiling analyses across multiple tumor types [21–23]. Such analyses may help to identify the commonalities and/or differences in molecular aberrations across cancer types and to assess therapeutic implications. A prime example is a pan-cancer analysis of SCNA patterns across 11 cancer types, which identified common patterns of SCNAs across cancer types and provided insight into the mechanisms of generation and functional consequences of cancer-related SCNAs [24]. We and others have used TCGA expression data to characterize cancer-related lncRNAs and their interaction maps [25–29] in multiple cancer types. As an increasing amount of cancer genomics data have become available, there remains a need to integrate various cancer genomics and clinical datasets to reveal the clinical phenotypes and driver potentials of lncRNAs in a pan-cancer manner.

To determine the landscape of lncRNA signatures across various cancer types, in the present study, we performed a large-scale lncRNA expression and SCNA profiling analysis in ~7000 clinical specimens from 15 different cancer types. We performed systemic comparison analysis of lncRNA profiles within and across cancer types and identified hundreds of dysregulated lncRNAs in each cancer and characterized 47 extensively dysregulated lncRNAs linked to at least 10 cancer types. We further confirmed our results by experimental q-PCR. We also identified many survival-related lncRNAs in each cancer which were independent of age and gender by multivariate Cox regression analysis. Finally, we analyzed the copy number variation of lncRNAs and suggested their oncogenic potentials.

## RESULTS

### Global lncRNA expression profiles across 15 cancer types

**Figure 1:**
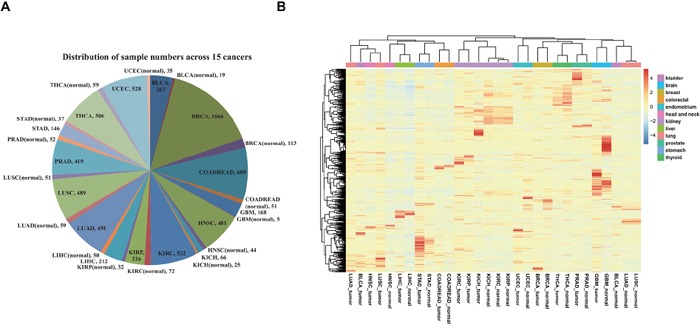
Overview of all specimens and lncRNAs **A.** Distributions of all specimens across 15 cancer types. **B.** Global expression profiles of 985 lncRNAs across cancer types. The tree displays their average expression values. The mean was computed from all specimens derived from the same type of normal tissue or tumor. Clustering was performed using the average and correlation metrics in the *pheatmap* function.

We first identified global expression signatures based on 985 lncRNAs that we observed across 6910 specimens, including matched normal tissues from 15 cancer types by performing unsupervised clustering analysis (Figure 1A; Supplementary Table 1). This pan-cancer clustering analysis revealed a highly tissue-specific lncRNA expression pattern for multiple cancer types (Figure 1B), which is in agreement with previous studies of normal human tissues [30, 31]. Out of the 15 cancer types that were analyzed, samples from 11 of the following cancer types tended to cluster together, irrespective of disease status (tumor or normal): breast cancer (BRCA), colon and rectum adenocarcinoma (COADREAD), glioblastoma multiforme (GBM), kidney carcinoma (KICH, KIRC, and KIRP), liver hepatocellular carcinoma (LIHC), prostate adenocarcinoma (PRAD), stomach adenocarcinoma (STAD), thyroid cancer (THCA), and uterine corpus endometrial carcinoma (UCEC). However, bladder urothelial carcinoma (BLCA), head and neck squamous cell carcinoma (HNSC), and lung carcinoma (LUAD and LUSC) presented significantly different signatures between tumor tissues and matched normal tissues, which suggests that these lncRNAs have potential to distinguish between these tissue types. Moreover, we discovered that many different tumor types—including BLCA, HNSC, LUAD and LUSC—showed similar signatures to each other, which suggests that a common expression of some lncRNAs may be ubiquitous in cancers.

### Identification of dysregulated lncRNAs within each individual cancer type

**Figure 2:**
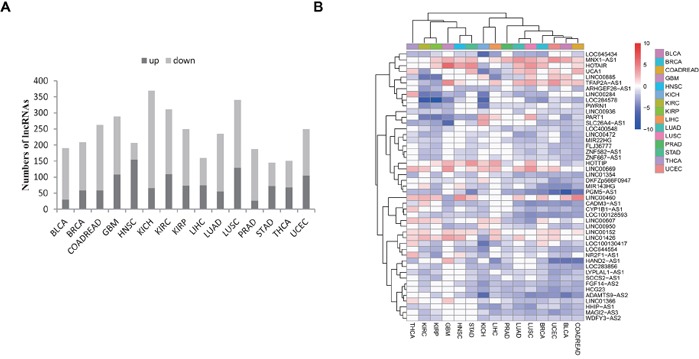
Identification of dysregulated lncRNAs in cancer **A.** The bar plot shows the numbers of dysregulated lncRNAs in each cancer type. The dark color represents up-regulation whereas the light color represents down-regulation. **B.** 47 lncRNAs show more pervasive patterns of dysregulation in ≥2/3 (10) tumor types. The tree displays their fold change levels after log2 transformation. Clustering was performed using the average and correlation metrics in the *pheatmap* function.

We subsequently identified lncRNAs that are differentially expressed between tumors and normal tissues within each of the 15 cancer types analyzed. Using an FDR<0.05 and a fold change>2 as the threshold, we identified significantly dysregulated lncRNAs for each cancer type (Figure 2A; Supplementary Table 2). Of these, we identified 145 significantly dysregulated lncRNAs in STAD (with a minimum number), and 369 lncRNAs in KICH (with a maximum number). Interestingly, across 15 cancer types, we discovered that more lncRNAs tended to be down-regulated than up-regulated (median 16.3% vs 7.3%). Our results included some well-known cancer-associated lncRNAs such as HOTAIR [32], PCA3 [33], PCAT1 [34], and CRNDE [35]. These lncRNAs demonstrated a similar pattern of dysregulation as previous studies for the specific cancer types in our study, which suggests the robustness of our approach. We also identified many novel dysregulated lncRNAs. For example, ANKRD34C-AS1 was found to be markedly down-regulated in GBM (~38-fold, FDR=4.62e-15), whereas UNC5B-AS1 was up-regulated in THCA (~17-fold, FDR=3.52e-22), and TTC21B-AS1 was up-regulated in KIRC (~92-fold, FDR=2.11e-30).

### Identification of commonly dysregulated lncRNAs across multiple cancer types

We further cross-compared the dysregulated lncRNAs that were identified from each cancer type. This cross-comparison identified 651 (of 811) lncRNAs that were dysregulated across at least two cancer types (Supplementary Table 2), which indicates a more common dysregulation pattern among multiple cancer types. For example, the lncRNAs LOC100128593 and PGM5-AS1 showed the most pervasive down-regulation in 13 cancer types; several well-characterized lncRNAs such as HOTAIR [32, 36], H19 [37–39] and PVT1 [16, 40, 41], also showed dysregulation in at least nine different cancer types. An expression signature consisting of 47 commonly dysregulated lncRNAs in over 2/3 of the cancer types analyzed (≥10) is depicted in Figure 2B. It is interesting that most of these lncRNAs were down-regulated as opposed to up-regulated across the 15 cancer types. The identification of commonly dysregulated lncRNAs across multiple cancer types indicates that these lncRNAs are possibly involved in the common and fundamental pathways of human tumorigenesis.

### Experimental validation of lncRNA dysregulation by q-PCR

**Figure 3:**
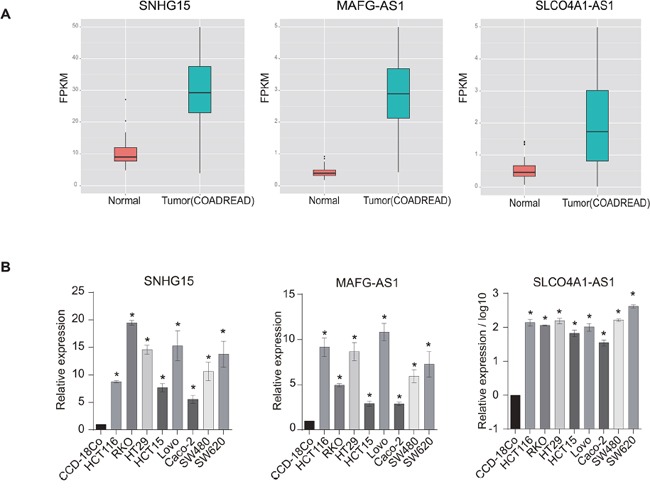
Experimental validation of dysregulated lncRNAs in colorectal cancer (CRC) cell lines **A.** The box plot shows three lncRNAs that are significantly up-regulated in CRC relative to normal tissues according to RNA-Seq data. **B.** Three randomly selected up-regulated lncRNAs were validated by q-PCR across eight CRC cell lines (p<0.05).

To confirm the alterations we observed for the above-mentioned lncRNAs, which were identified from the RNA-Seq data, we conducted quantitative real-time PCR (q-PCR). Based on the availability of cancer cell lines as well as corresponding normal controls (see Materials and Methods), we performed q-PCR validation for COAD/READ. We randomly selected three lncRNAs that were significantly up-regulated in tumors compared with normal tissues according to the above RNA-Seq data analysis for experimental validation (SNHG15, MAFG-AS1 and SLCO4A1-AS1) (Figure 3A). In agreement with these findings, the q-PCR results confirmed the changes in expression patterns for the three lncRNAs in all eight CRC cell lines (compared with normal colon cells; Figure 3B). This suggests the reliability of our RNA-Seq analysis.

### Evaluation of the prognostic power of lncRNAs

**Figure 4:**
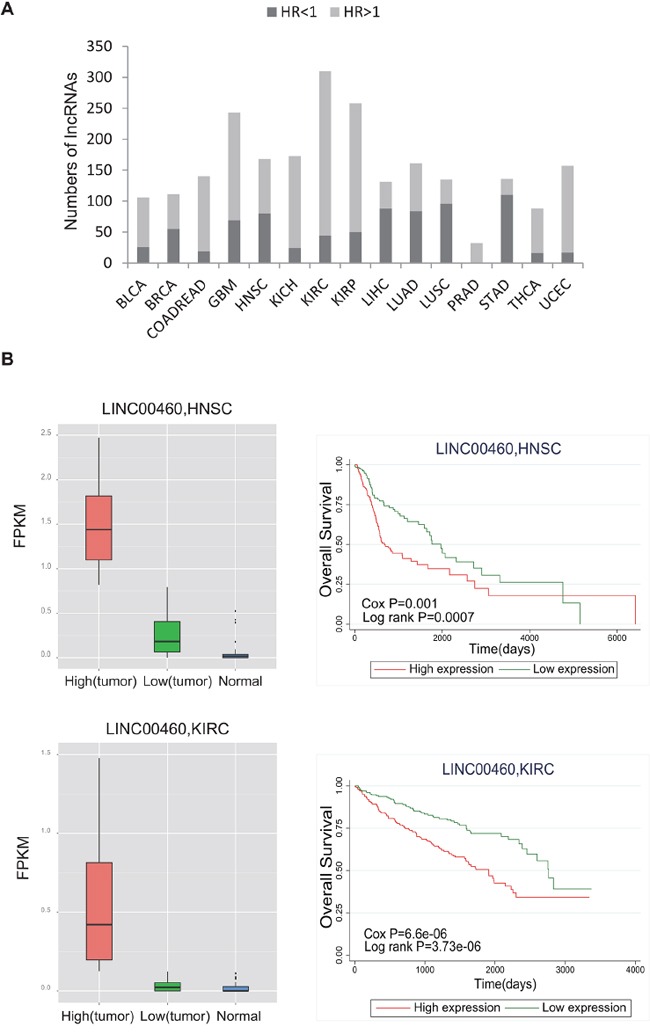
Evaluation of the prognostic power of lncRNAs **A.** The bar plot shows the number of significantly prognostic lncRNAs in each cancer type. The dark color represents HR<1, and the light color represents HR>1. **B.** The box plot shows differential expression of LINC00460 between tumor and normal tissue for HNSC and KIRC. Survival curves demonstrate that higher expression of LINC00460 is associated with worse survival. LINC00460 expression values were dichotomized into low and high expression groups using the within-cohort median expression value as a cutoff. Red curves represent the high expression group, and green curves represent the low expression group. Survival curves were plotted using Kaplan–Meier methods, and statistical significance was assessed using log-rank tests.

We assessed the prognostic significance of lncRNAs by multivariate Cox regression analysis with gender and age as covariates. With a threshold of p<0.05, we identified survival-related lncRNAs in each cancer type (ranging from 32 to 310 in number; Figure 4A; Supplementary Table 3). Of these, 32 lncRNAs (out of 985) were significant prognostic indicators for PRAD, and 310 lncRNAs were prognostic indicators for KIRC (Supplementary Figure 1A and 1B). Further analysis revealed that 77.4% of these prognostic lncRNAs appeared in at least two different cancers, which suggests their potential power as general prognostic biomarkers. For example, LOC90768 was found to be significantly associated with overall patient survival in nine different cancer types. Moreover, lncRNA expression tended to be associated with an increased risk of cancer (hazard ratio (HR)>1; Figure 4A). For instance, for LINC00460, which is up-regulated in HNSC and KIRC relative to normal tissues, higher expression is significantly correlated with worse survival (Figure 4B).

### Somatic copy number analysis reveals oncogenic potentials of lncRNAs

**Figure 5:**
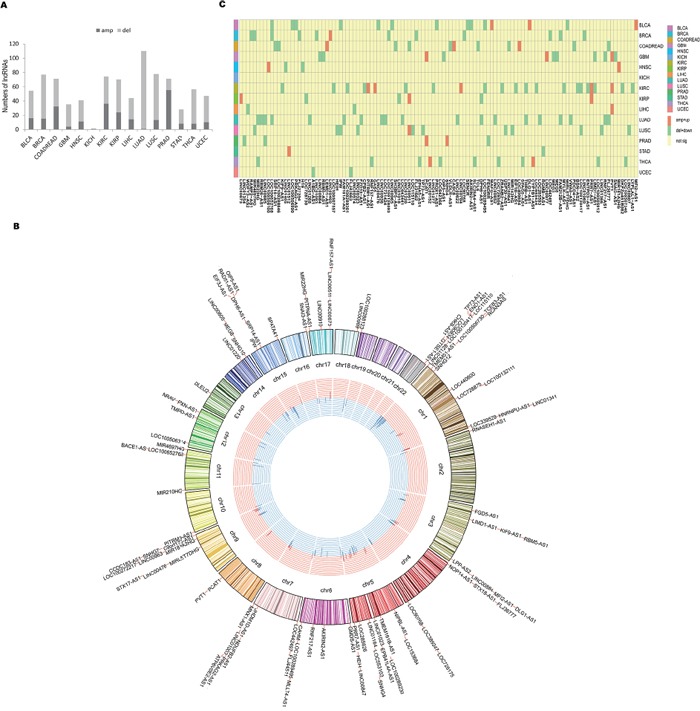
Somatic copy number analysis of lncRNAs **A.** The bar plot shows the number of SCNA-containing lncRNAs with positive correlation between expression and copy number in each cancer type. The dark color represents amplified lncRNAs whereas the light color represents deleted lncRNAs. **B.** The Circos plot shows the distribution of 102 SCNA-containing lncRNAs with positive correlation between expression and copy number within the genome and across multiple cancer types (≥3 cancer types). The outermost track represents chromosomes. Histograms located in the innermost track represent the number of cancer types associated with the lncRNAs. Red represents amplification, and blue represents deletion. The distance between any two curves represents one cancer type. **C.** The heatmap shows 116 dysregulated lncRNAs are strikingly genomic altered across 15 cancer types. Yellow represents values that are not significant; light green represents deletion and down-regulation; orange represents amplification and up-regulation.

It has been suggested that genes with causal roles in oncogenesis are often located in the SCNAs that are frequently altered across tumors [16–18, 24, 42]. Thus, we mapped lncRNA genes to regions of recurrent SCNA regions identified from 15 cancer types. Among 985 lncRNAs, we found 85.7% of the lncRNAs were located in recurrent SCNA regions and approximately half of them presented significantly positive correlation with copy number (FDR<0.05; Figure 5A; Supplementary Table 4). This demonstrated SCNA may be an important mechanism for disrupting expression of lncRNAs. Notably, most of these lncRNAs were located in deleted regions rather than in amplified regions (median 3.8% vs 1.1%). Moreover, it was interesting that many of these lncRNAs were extensively expressed in multiple cancer types (Figure 5B). For example, PVT1, a lncRNA that was consistently amplified and expressed in four different cancer types, is located at the chromosome site 8q24. This region is commonly amplified in multiple human cancers [16, 40, 41]. Another example is CHKB-AS1, the deletion of which was consistently present in 10 different cancer types in our study. Strikingly, 116 dysregulated lncRNAs were markedly genomic altered across 15 cancer types (Figure 5C; Supplementary Table 5), suggesting their oncogenic potentials. For example, PVT1 was significantly amplified and up-regulated in GBM, KIRC and LIHC; PCAT1 was significantly amplified and up-regulated in GBM and PRAD.

## DISCUSSION

The dysregulated expression patterns of lncRNAs, as well as their biological relevance, have been reported in an increasing number of cancer types [32, 43–46]. However, most of these studies have been restricted to single tumor types and have used a modest number of samples. In the present study, we integrated multiple types of data, including expression profiling data, SCNA data and clinical information for ~7000 clinical samples from 15 cancer types and then conducted a large-scale pan-cancer analysis. We identified differently expressed lncRNAs within and cross-cancer. We also evaluated the prognostic power of lncRNAs and identified a group of lncRNAs as general biomarkers that may simultaneously predict the prognosis of multiple cancers. Moreover, we also suggested potential oncogenic lncRNAs via an integrative analysis of the association between the expression of lncRNAs and SCNAs.

We found that lncRNAs tend to be down-regulated rather than up-regulated in cancers compared to normal tissues, suggesting a major tumor-suppressive potential. We reported many novel, markedly dysregulated lncRNAs that have not been reported by previous studies. For example, expression levels for TTC21B-AS1 are specifically increased ~ 92-fold in KIRC compared with normal tissues. These cancer-specific and dysregulated lncRNAs may act as potential novel clinical biomarkers. Cross-cancer analysis identified a group of lncRNAs that was commonly dysregulated among multiple cancer types. For example, PVT1 was found to be up-regulated in nine different cancer types. PVT1 has been reported to be an oncogene in colorectal cancer (CRC), the knockdown of which could inhibit cell proliferation and the invasion capabilities of CRC cells via TGF-beta signaling and apoptotic pathways [47]. The lncRNA HOTAIR was extensively up-regulated in eight different cancer types. HOTAIR was originally found to be up-regulated in breast and colon cancers and was correlated with poor prognosis based on its interaction with PRC2 and it's epigenetic regulation of metastasis-related genes [32, 36]. The common dysregulation of these lncRNAs in multiple cancer types suggests that these lncRNAs may be involved in the fundamental pathways that are important to the initiation and progression of various cancers. These lncRNAs may therefore lead to the discovery of new drug targets [48].

Given the widespread change in lncRNA expression across different cancer types, is there a general cancer-related mechanism that effects lncRNA biosynthesis? Indeed, aberrant expression of some molecules similarly tend to the same cause across different cancer types. For example, oncogenic PVT1 are overexpressed across multiple cancer types because of amplification [16]; TERT presented increased expression across multiple cancer types because of recurrent somatic mutations in the promoter [49]; MEG3 are underexpressed in tumors mainly due to promoter silencing by hypermethylation [50]. However, expression of other molecules can be controlled by multiple factors. An example, HOTAIR, overexpressed in multiple cancer types because of effects of c-Myc, TGF-β or other regulatory factors [51]. Thus, in this instance, it is hard to say there is a general cancer-related mechanism that effects lncRNA biosynthesis. If a lncRNA, we argue, is certainly need for cancer development, it will always be active via multiple selective mechanisms such as copy number variation or mutation.

We also assessed the associations between the expression of lncRNAs and patient survival and identified a group of lncRNAs with significant prognostic value. Unexpectedly, many of these prognostic lncRNAs were linked to multiple cancer types. For example, LOC90768 showed prognostic significance in nine different cancer types, and HR values indicated that higher expression of this lncRNA leads to poor survival in seven cancer types. Another example is LINC00460, the expression of which predicts worse patient survival in both HNSC and KIRC. This lncRNA was also found to be up-regulated in tumors relative to normal tissues in these two cancer types. Together, these findings suggest the oncogenic or risk-promoting role of LINC00460 in tumorigenesis. Further validation in additional independent data sets may provide a wider perspective on the prognostic power of these lncRNAs.

In addition, through integrating SCNAs and expression data, we revealed sets of lncRNAs that were strikingly genomic altered across 15 cancer types. Meanwhile, their expressions were significantly positive correlation with gene copy number. Strikingly, 116 of them show significant dysregulation in tumor relative to normal tissues. Previous studies have identified suchlike lncRNAs as drivers such as FAL1[17] and PCAN-R1/2[18]. These data may be valuable for investing the function of lncRNAs and lay the groundwork for future experiment studies.

Our study has unique advantages over related studies [29, 52]. First, more cancer types were included in this study, which was two times as many as previous studies. Thus, we revealed many commonly dysregulated lncRNAs among cancer types instead of the few reported by previous studies. This greatly increased our outstanding for the dysregulation of lncRNAs across cancer types. Second, we used the expression level generated by the Rsubread pipeline that produced more consistent expression levels across replicate samples than the TCGA pipeline [53]. In addition, we applied limma package for differential expression analysis, this method has been demonstrated as the best practice among various of software packages in RNA-seq studies [54]. Third, our results illuminated the prognostic landscape of lncRNAs across cancer types, and suggested potential prognostic biomarkers for further investigation and clinical translation.

In conclusion, we performed a large-scale pan-cancer analysis of lncRNAs via an integration of the matched expression profiles, the SCNA profiles, and the clinical information for ~7000 specimens across 15 different cancer types. We identified a group of clinically relevant lncRNAs that may act as potential drivers and biomarkers for cancer typing, prognosis and targeted therapy. Our study provided a landscape of lncRNAs in cancer and may accelerate the pace of experimental or clinical studies of lncRNAs in human cancer.

## MATERIALS AND METHODS

### RNA-Seq and the collection and processing of clinical data

For the 15 cancer types we analyzed, summarized expression values and clinical information (Supplementary Table 6) for all available tumors and for at least 5 normal samples per cancer type were downloaded from NCBI's Gene Expression Omnibus (GEO) via accession number GSE62944[53]. These data include expression values for lncRNA and protein-coding genes. In creating this data set, raw RNA-Sequencing reads and the corresponding clinical information for a total of 6910 specimens from 15 cancer types had been downloaded via https://cghub.ucsc.edu and the TCGA Data Portal [55]. The reads had been processed and normalized using the Rsubread package (version 1.14.2) [56] and aligned to the UCSC hg19 reference genome. The featureCounts function was used to summarize the gene expression values as integers. These summarized gene values were then normalized to FPKM values. To accurately annotate lncRNA, we used our lncSeeker pipeline [57] to filter the lncRNAs downloaded from Refseq database. The filter steps were briefly described as follow: (1) remove the transcripts with length less than 200 nt. (2) Transcripts were excluded from further consideration if they overlap with non-lincRNA annotation, such as protein-coding genes, pseudogenes and other small RNAs. (3) Coding potential filter. Transcripts with ORF <100 aa, ORF coverage less than 30% and txCdsPredict score <800 were classified as lncRNAs. (4) Known protein domains filter. Transcript was mapped to unitRef90 database with BlastX and transcripts with E-value < 1E–30 were removed. Moreover, Transcripts with a Pfam hit showing both the full sequence E-value < 1E–5 and the single best domain E-value < 1E–5 were removed. In total, 985 known lncRNAs were measured in our study (Supplementary Table 7).

### Data analysis and statistical methods

Unsupervised clustering was performed using average and correlation options in the “pheatmap” software package in R (version 3.2.0). Significant differences in the expression of lncRNAs between any two comparison groups were defined using the “limma” software package in the same R version. A false discovery rate (FDR) value of less than 0.05 and a fold change (FC) of greater than 2 were defined as statistically significant. The correlation between lncRNA expression and the overall survival of the patients was assessed by both Cox regression analysis and the Kaplan-Meier estimation method. In the Cox regression analysis, the lncRNA was evaluated as a continuous variable with age and gender as additional covariables. For the Kaplan-Meier estimates, we defined the high-expression groups and low-expression groups using the median lncRNA expression value as a cut-off point. Two-group survival curves were assessed for significance using a log-rank test.

### Somatic copy number analysis of lncRNAs

SCNA regions identified using the GISTIC 2.0 [58] software were downloaded from the Firebrowse website (https://confluence.broadinstitute.org/display/GDAC/Dashboard-Analyses) created by the Broad Institute. We identified “wide peaks” of the focally amplified or deleted regions defined by GISTIC2.0; the lncRNAs within these regions were identified using BEDTools [59]. The correlation between copy number values and the corresponding gene expression levels was estimated using R (version 3.2.0, Pearson correlation), and p-values were adjusted using an FDR correction.

### q-PCR validation of the dysregulated lncRNAs in cell lines

Human colon carcinoma cell lines HCT116, RKO, HT29, HCT15, Lovo, Caco-2, SW480 and SW620 were obtained from the Cell Bank of the Type Culture Collection of the Chinese Academy of Sciences (Shanghai, China) and were cultured according to the manufacturer's instructions. The CCD-18Co normal human colon cell line was a gift from Dr Yanyun Zhang (The Institute of Health Sciences, SIBS, CAS / SJTUSM, Shanghai, China) and was cultured in MEM medium (Gibco) supplemented with 0.11 g/L Sodium Pyruvate (Sigma), 10% FBS and 1% penicillin-streptomycin (Gibco) at 37°C in a humidified atmosphere with 95% air and 5% CO2.

Total cellular RNA was extracted using TRIzol (Invitrogen) according to the manufacturer's instructions. cDNA was then synthesized using a PrimeScript™ RT reagent Kit with gDNA Eraser (TaKaRa). q-PCR was performed with SYBR Premix Ex Taq II (Ti RnaseH Plus)(TaKaRa) in a Roche LightCycler480 System with three biological replicates. The comparative Ct Method (ΔΔCT Method) was used to quantify the relative gene expression; β-actin was used for normalization. The gene-specific primers are listed in Supplementary Table 8.

## SUPPLEMENTARY FIGURE AND TABLES














