# Supplementary material for: Integrative analysis reveals clinical phenotypes and oncogenic potentials of long non-coding RNAs across 15 cancer types
Source: Oncotarget. 2016 Apr 27;7(23):35044–55. doi: 10.18632/oncotarget.9037 (PMC5085208; doi:10.18632/oncotarget.9037)
Supplement: Supplementary file 1 [file oncotarget-07-35044-s001.pdf]

SUPPLEMENTARY FIGURE AND TABLES

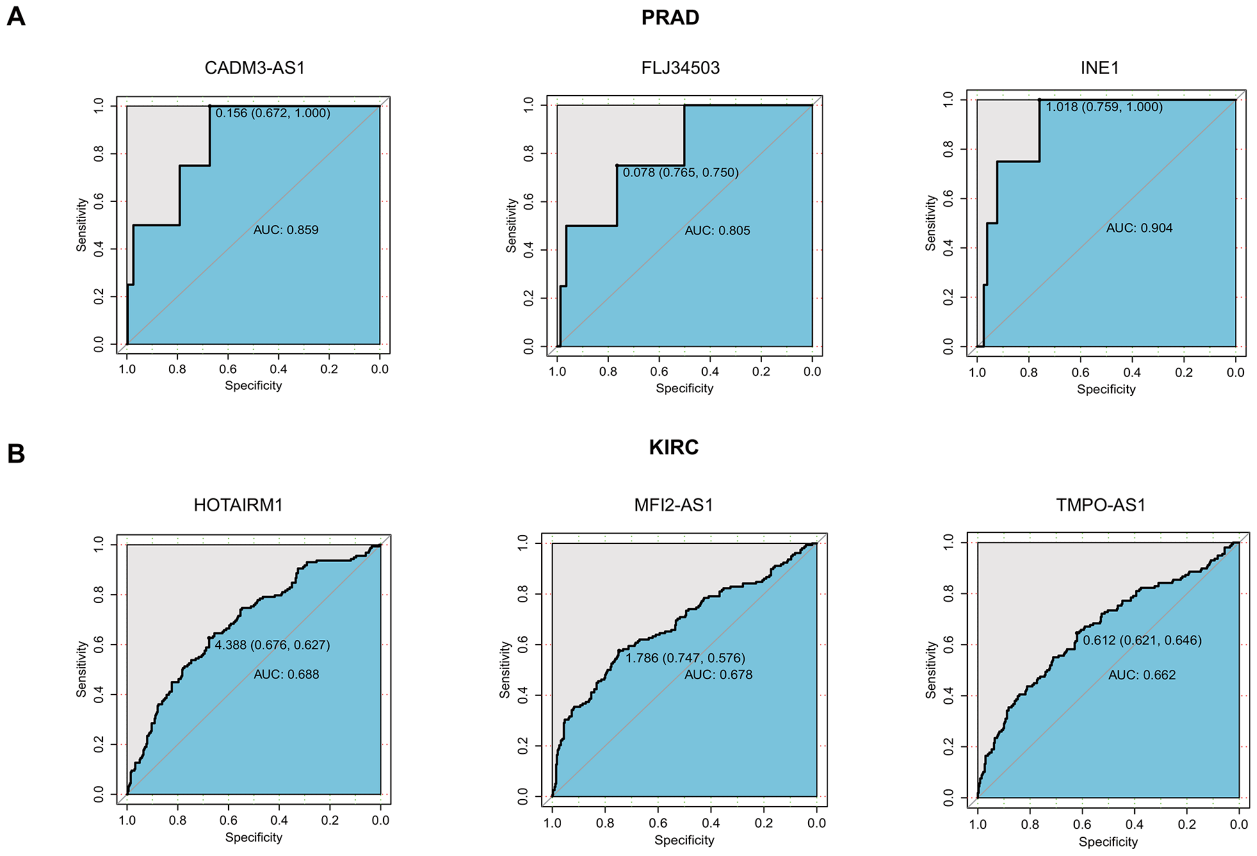

Supplementary Figure S1: ROC analysis of several representative prognostic lncRNAs. A. ROC curves of three most significant prognostic lncRNAs in PRAD. **B.** ROC curves of three most significant prognostic lncRNAs in KIRC.

Supplementary Table S1: A summary of all specimens across 15 cancer types

Distributions of all specimens across 15 cancer types		
**Type**	**Tumor**	**Normal**
Bladder Urothelial Carcinoma(BLCA)	267	19
Breast invasive carcinoma(BRCA)	1066	113
Colon and rectum adenocarcinoma(COADREAD)	609	51
Glioblastoma multiforme(GBM)	168	5
Head and Neck squamous cell carcinoma(HNSC)	481	44
Kidney Chromophobe(KICH)	66	25
Kidney renal clear cell carcinoma(KIRC)	532	72
Kidney renal papillary cell carcinoma(KIRP)	226	32
Liver hepatocellular carcinoma(LIHC)	212	50
Lung adenocarcinoma(LUAD)	491	59
Lung squamous cell carcinoma(LUSC)	489	51
Prostate adenocarcinoma(PRAD)	419	52
Stomach adenocarcinoma(STAD)	146	37
Thyroid carcinoma(THCA)	506	59
Uterine Corpus Endometrial Carcinoma(UCEC)	528	35

Supplementary Table S2: Dysregulated lncRNAs across 15 cancer types

See Supplementary File 1

Supplementary Table S3: Survival-associated lncRNAs across 15 cancer types

See Supplementary File 2

Supplementary Table S4: The lncRNAs that expressions are positively correlated with gene copy number and located in recurrent SCNA regions across 15 cancer types

See Supplementary File 3

Supplementary Table S5: 116 dysregulated lncRNAs are strikingly genomic altered across 15 cancer types

See Supplementary File 4

Supplementary Table S6: Summarized clinical information of analyzed 15 cancer types

See Supplementary File 5

Supplementary Table S7: A summary of the 985 lncRNAs that were analyzed in the present study

See Supplementary File 6

Supplementary Table S8: Primers used for q-PCR

Primer List	
**Primer**	**Sequence(5’-3’)**
SNHG15-F	GCCTGCCTGTGTTAATAC
SNHG15-R	AATTCCTGACTCCTTCCA
SLCO4A1-AS1-F	GATGAACGCAACTCTGAA
SLCO4A1-AS1-R	CGTCTGTTCCTGATTCTTC
MAFG-AS1-F	GTGTTCCGTGGTCAGTGA
MAFG-AS1-R	CAAGTGTCCTGGGCTCTC
β-actin-F	GAAACTACCTTCAACTCCATC
β-actin-R	CGAGGCCAGGATGGAGCCGCC
